# Aortic Dissection Masquerading as Pneumonia: A Case Report of an Atypical Presentation

**DOI:** 10.7759/cureus.65930

**Published:** 2024-08-01

**Authors:** Gurjot Singh, Shubam Trehan, Didar Singh, Kanishka Goswami, Rajpreet S Arora

**Affiliations:** 1 Internal Medicine, Saint John Hospital, Southern Illinois University, Springfield, USA; 2 Hospital Medicine, Springfield Clinic, Springfield, USA; 3 Internal Medicine, Springfield Memorial Hospital, Southern Illinois University, Springfield, USA

**Keywords:** endovascular intervention, cardiovascular emergency, diffuse abdominal pain, atypical chest pain, misdiagnosis, atypical presentation, aortic dissection

## Abstract

Aortic dissection is a critical and life-threatening condition that can present with atypical symptoms, often leading to misdiagnosis and delayed treatment. The report presents a case of a 65-year-old male who initially exhibited fever, right-sided chest pain, and a productive cough, resulting in an initial diagnosis of pneumonia. Despite empirical antibiotic therapy, his symptoms persisted, prompting further investigation. A computed tomography (CT) scan ultimately revealed a Type B aortic dissection. The patient was then transferred to a specialized tertiary care facility for successful endovascular intervention. This case underscores the importance of considering aortic dissection in patients presenting with persistent, atypical symptoms that do not respond to standard treatments, such as unexplained fever and chest pain. It highlights the crucial role of advanced imaging techniques, such as CT scans, in achieving an accurate and timely diagnosis. Clinicians must maintain a high index of suspicion and ensure prompt referral to specialized centers to improve patient outcomes in this potentially fatal condition.

## Introduction

Aortic dissection is a severe and life-threatening condition that involves a tear in the intimal layer of the aorta, allowing blood to flow between the layers of the aortic wall and causing them to split. This medical emergency necessitates immediate diagnosis and intervention to mitigate high mortality rates. The classic presentation of aortic dissection includes sudden, severe chest pain, often described as tearing or ripping, radiating to the back. However, there are cases where the presentation is atypical, complicating the diagnostic process and potentially delaying appropriate treatment [1-2].

The incidence of aortic dissection is estimated to be between 2.6 and 3.5 cases per 100,000 person-years, making it a relatively rare but highly dangerous condition [3]. Despite its rarity, aortic dissection is one of the most severe cardiovascular emergencies due to its potential for rapid deterioration and significant mortality.

Key risk factors for aortic dissection include hypertension, which is the most significant risk factor, present in about 75% of cases. Chronic high blood pressure can weaken the aortic wall, making it more susceptible to dissection [4]. Atherosclerosis, characterized by the buildup of plaques in the arterial walls, also contributes to the weakening of the aorta, increasing the risk of dissection [5]. Genetic conditions such as Marfan syndrome, Ehlers-Danlos syndrome, and bicuspid aortic valve significantly raise the risk due to underlying connective tissue abnormalities [6]. Age and sex are additional factors, with aortic dissection being more common in men, particularly those aged 60 to 80 years [2]. Trauma, including physical trauma to the chest or high-impact injuries, can precipitate aortic dissection. Iatrogenic factors, such as surgical or catheter-based interventions involving the aorta, also pose a risk. Substance abuse, especially cocaine use, is linked to acute increases in blood pressure and the subsequent risk of dissection [7]. Additionally, although rare, pregnancy can elevate the risk of aortic dissection, particularly in women with underlying connective tissue disorders [8].

While the typical presentation of aortic dissection involves the acute onset of severe chest or back pain, described as sharp, tearing, or ripping, atypical presentations are not uncommon and present significant diagnostic challenges. Patients with atypical presentations may exhibit symptoms that mimic other conditions, such as myocardial infarction, stroke, or, in rare instances, pneumonia. For example, a study by Nienaber et al. found that 15% of patients initially presented with symptoms resembling stroke rather than classic chest pain [9]. Such misleading symptoms can result in misdiagnosis and delayed treatment, thereby increasing the risk of adverse outcomes [10].

To address these diagnostic challenges, advanced imaging techniques such as CT, MRI, and transesophageal echocardiography (TEE) are crucial for confirming the diagnosis of aortic dissection, given their high sensitivity and specificity; CT scans, in particular, are often the first-line imaging modality due to their widespread availability and rapid acquisition time, which is critical in emergency settings.

This report discusses a case where a 65-year-old male was initially misdiagnosed with pneumonia due to atypical symptoms, ultimately revealing a Type 1 DeBakey aortic dissection. This case underscores the diagnostic challenges and the importance of considering aortic dissection in differential diagnoses. Comprehensive evaluation and prompt referral to specialized centers for appropriate intervention are paramount to improving patient outcomes in such complex cases.

## Case presentation

A 65-year-old male with no known cardiovascular history was admitted to the emergency department presenting with symptoms: persistent mild-grade fever from the past week, right-sided chest pain, and a productive cough persisting for four days. On examination, the patient was alert, cooperative, and oriented. His vital signs included a pulse rate of 96 beats per minute with a regular rhythm, a respiratory rate of 22 breaths per minute, and a temperature of 98°F. Blood pressure readings were 130/60 mmHg in the right arm and 135/64 mmHg in the left arm. Oxygen saturation was 96% on room air, with no signs of cyanosis, jaundice, clubbing, or significant lymphadenopathy.

Auscultation of the respiratory system revealed coarse crepitations bilaterally, with mildly reduced air entry on the right side. The cardiovascular examination was notable for normal S1 and S2 heart sounds, accompanied by a long early diastolic murmur. Neurological examination findings were normal, and the abdomen was soft with no palpable organomegaly. Laboratory investigations showed a hemoglobin level of 11.8 g/dL (normal range 12-15 g/dL), a total leukocyte count of 12,400/cumm (normal range 4,000-10,000/cumm) with a predominance of neutrophils (80%), and other differential counts within normal limits. The erythrocyte sedimentation rate (ESR) was elevated at 56 mm/hr (Table 1). Biochemical analyses revealed mildly deranged liver function tests, with elevated serum glutamic-oxaloacetic transaminase (SGOT) and serum glutamic-pyruvic transaminase (SGPT) levels. Electrolyte analysis indicated hyponatremia with a sodium level of 127 mEq/L (normal range 136-145 mEq/L (Table 2).

**Table 1:** The patient's hematology lab reports ESR: erythrocyte sedimentation rate; CRP: C-reactive protein

Lab reports	Results	Reference range
White blood cell count (1000/cumm)	12.4	11-Apr
Platelet count (1000/cumm)	191	150-450
Red blood cell count (million/uL)	4.89	4.0-5.1
Hemoglobin (g/dL)	11.8	14-Dec
Mean corpuscular volume (fL)	78.4	80-100
Mean corpuscular hemoglobin (pg)	27.5	27.5-33.2
Mean corpuscular hemoglobin concentration (gm/dL)	33.4	33.4-35.5
Neutrophils (%)	80	40-80
Lymphocytes (%)	10	20-40
Eosinophils (%)	6	6-Jan
Monocytes (%)	4	10-Feb
Basophils (%)	0	0-1
ESR (mm/hr)	56	0-20
CRP (mg/dL)	80	<0.3

**Table 2:** The patient's biochemistry lab reports

Biochemistry tests	Results	Reference range
Blood urea nitrogen (mg/dL)	30	15-40
Creatinine (mg/dL)	0.9	0.5-1.3
Glomerular filtration rate (mL/min/1.73 sq mm)	96	90-120
Total bilirubin (TBI) (mg/dL)	0.8	0.2-1.0
Direct bilirubin (DBI) (mg/dL)	0.2	<0.2
Indirect bilirubin (IBI) (mg/dL)	0.6	0.2-0.8
Aspartate aminotranferase (U/L)	41	15-37
Alanine aminotransferase (U/L)	63	14-59
Alkaline phosphatase (U/L)	91	<98
Lactate dehydrogenase (LDH) (U/L)	500	<250
Total protein (TP) (gm/dL)	5.9	6.4-8.3
Albumin (ALB) (gm/dL)	3.5	3.2-4.5
Sodium (mEq/L)	127	135-145
Potassium (mEq/L)	4.3	3.5-5.0

**Figure 1:**
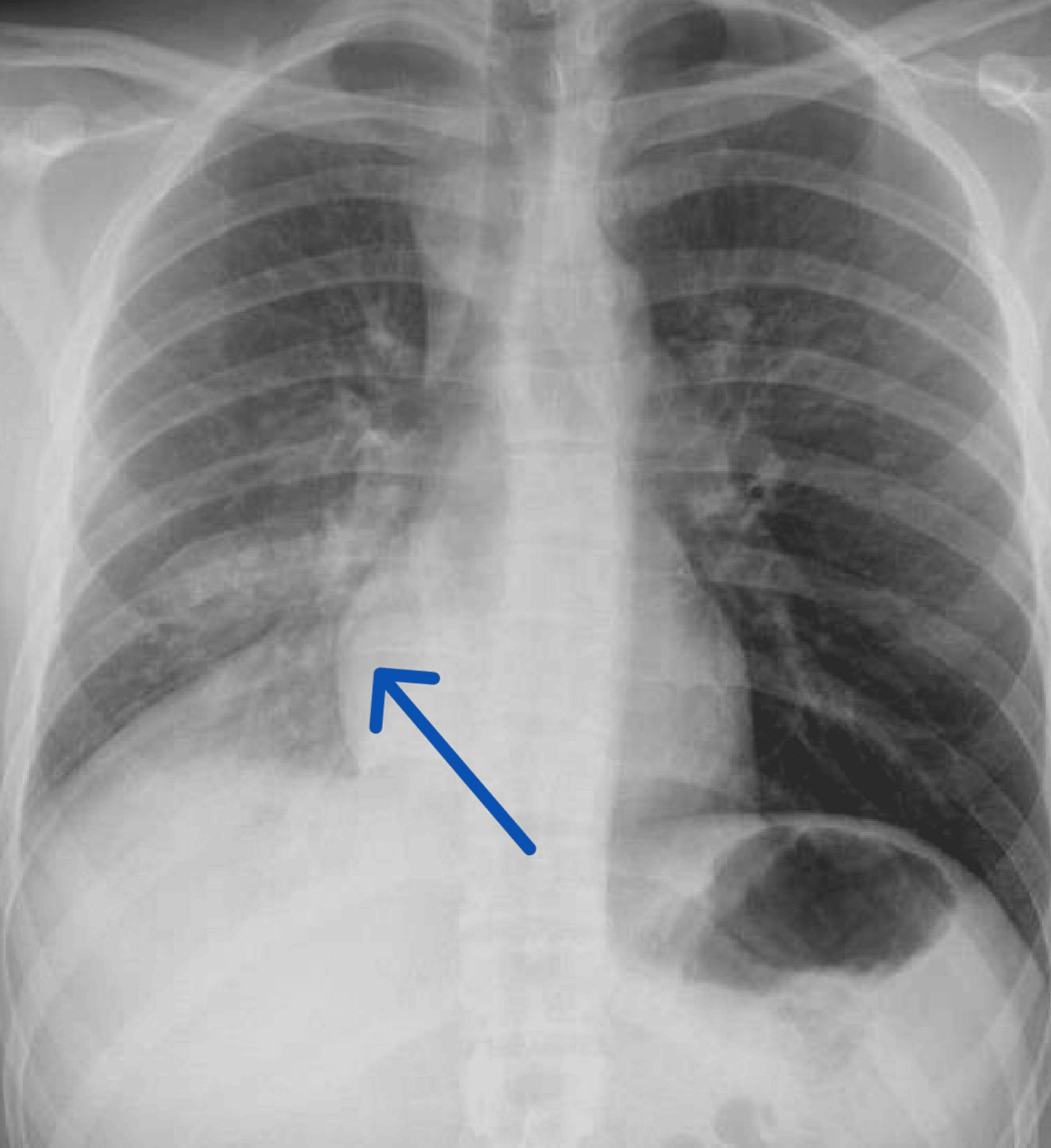
Chest X-ray (anteroposterior view) revealed right lower lobe opacity.

Chest X-ray findings showed opacity in the right mid and lower zones, with minimal blunting of the costophrenic angle (Figure 1).

Empirical antibiotic therapy was initiated; however, the patient’s clinical status did not improve. He subsequently developed intermittent abdominal pain. The persistence of fever and the onset of abdominal discomfort, combined with the lack of response to antibiotic treatment, prompted further investigation.

**Figure 2:**
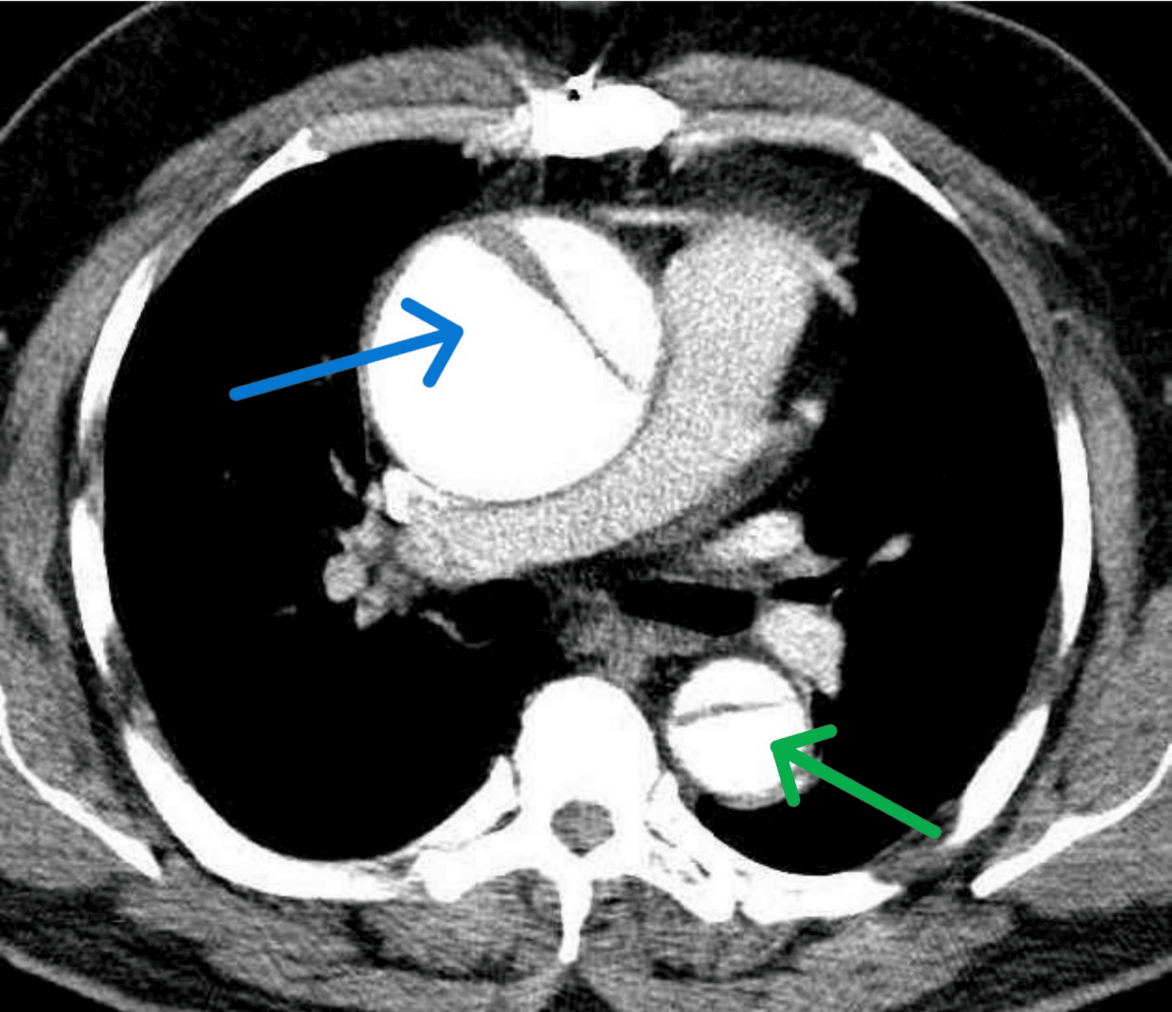
Chest CT (axial view) revealed ascending aorta dissection (blue arrow) and descending thoracic aorta dissection (green arrow).

Given the atypical evolution of his symptoms, a CT scan of the chest and abdomen was performed. The scan revealed a Type I DeBakey aortic dissection, a dissection extending from the ascending aorta to the descending thoracic aorta (Figure 2).

Notably, the patient exhibited no signs of hemodynamic instability, a common feature in many acute dissection cases. Following confirmation of the diagnosis, the patient was promptly transferred to a tertiary care facility for definitive management, which involved endovascular intervention.

## Discussion

Aortic dissection, although rare, represents one of the most severe and rapidly fatal conditions affecting the cardiovascular system. This case underscores the diagnostic challenges faced by clinicians, particularly with atypical presentations. Typically, aortic dissection presents with acute, severe chest or back pain, often described as tearing or ripping in nature. However, the spectrum of presentations can be broad, and atypical symptoms such as those observed in this case-fever, right-sided chest pain, and productive cough-can lead to misdiagnosis. The absence of classical symptoms like excruciating chest pain and the presence of misleading respiratory symptoms posed a significant diagnostic challenge. In a study by Hagan et al. [2], approximately 10% of patients with aortic dissection presented without chest pain, with primary symptoms including neurological deficits, abdominal pain, or symptoms mimicking respiratory infections. This variability necessitates a high index of suspicion and comprehensive evaluation in patients with persistent, unexplained symptoms. Such misleading symptoms can result in misdiagnosis and delayed treatment, thereby increasing the risk of adverse outcomes [10].

The differential diagnosis of aortic dissection must be broad, particularly when the presentation is atypical. Conditions such as myocardial infarction, pulmonary embolism, and pneumonia must be considered and ruled out. In this case, the initial misdiagnosis of pneumonia delayed the identification of the underlying aortic dissection. Despite empirical antibiotic therapy, the patient's symptoms persisted, prompting further investigation. Literature indicates that atypical presentations can lead to significant diagnostic delays. For instance, Nienaber et al. [10] highlighted that transient ischemic attack symptoms in aortic dissection can be misleading, often leading to initial misdiagnosis as a stroke. 

Advanced imaging techniques are crucial in diagnosing aortic dissection, particularly when clinical presentation is atypical. CT, MRI, and TEE are the primary modalities used to confirm the diagnosis. In this case, a CT scan of the chest and abdomen was instrumental in identifying the dissection. Hagan et al. [2] emphasized that prompt imaging is essential for an accurate diagnosis. The International Registry of Acute Aortic Dissection (IRAD) study showed that imaging modalities like CT, MRI, and TEE have high sensitivity and specificity for detecting aortic dissection. The 2010 ACCF/AHA/AATS/ACR/ASA/SCA/SCAI/SIR/STS/SVM guidelines (a report by the American College of Cardiology Foundation/American Heart Association Task Force on Practice Guidelines, American Association for Thoracic Surgery, American College of Radiology, American Stroke Association, Society of Cardiovascular Anesthesiologists, Society for Cardiovascular Angiography and Interventions, Society of Interventional Radiology, Society of Thoracic Surgeons, and Society for Vascular Medicine) recommend the use of these advanced diagnostic tools in suspected cases of aortic dissection, highlighting their importance in facilitating an accurate diagnosis and ensuring timely intervention [2,11]. Computed tomography scans are particularly valuable due to their rapid acquisition time, making them essential in emergency settings.

Recent advancements in imaging technology, such as high-resolution CT and MRI, have further improved the ability to detect aortic dissections early. These advancements allow for better visualization of the aorta and the extent of the dissection, aiding in the prompt and accurate diagnosis of this life-threatening condition. Additionally, the use of contrast-enhanced CT angiography provides detailed images that help in planning the appropriate intervention strategy [10].

The management of aortic dissection depends on the type and extent of the dissection, as well as the patient's hemodynamic status. For Stanford Type B (Type 2) dissections, which involve the descending aorta, management may include medical therapy to control blood pressure and pain or endovascular techniques such as thoracic endovascular aortic repair (TEVAR) depending on the presence of complications. TEVAR involves the placement of a stent graft within the aorta to cover the dissection flap and reinforce the aortic wall. This minimally invasive approach reduces the risks associated with open surgery and allows for quicker recovery [11]. TEVAR is particularly advantageous in high-risk patients who may not tolerate open surgery. However, it requires precise imaging and expertise to ensure the correct placement of the stent graft and avoid complications such as endoleak, where blood continues to flow into the false lumen created by the dissection [11]. Pharmacological management in cases of aortic dissection includes the use of beta-blockers to reduce heart rate and blood pressure, thereby decreasing the stress on the aortic wall. Other antihypertensive agents, such as calcium channel blockers and angiotensin-converting enzyme (ACE) inhibitors, may also be used to achieve optimal blood pressure control. In acute settings, intravenous medications are preferred for their rapid onset of action [12].

In the present case, the patient underwent endovascular intervention, highlighting the importance of timely diagnosis and appropriate referral to specialized centers capable of performing such procedures. The absence of hemodynamic instability, despite the presence of aortic dissection, is notable. It highlights that a high degree of vigilance is necessary even in the absence of classical signs of hemodynamic compromise. 

## Conclusions

This case underscores the critical importance of considering aortic dissection in the differential diagnosis of persistent, unexplained symptoms, even when initial presentations mimic more common conditions such as pneumonia. The patient's atypical symptoms, including fever and a productive cough, led to an initial misdiagnosis, delaying the recognition and appropriate treatment of the underlying aortic dissection. This highlights the necessity for advanced imaging techniques like CT, MRI, and TEE to achieve timely and accurate diagnosis. Clinicians must maintain a high index of suspicion, particularly in patients with significant risk factors such as hypertension, advanced age, or genetic predispositions. A heightened awareness and systematic approach to diagnosing and managing this potentially fatal condition are imperative for enhancing patient survival and quality of care.
